# Impact of Serum Uric Acid Level on Systemic Endothelial Dysfunction in Patients with a Broad Spectrum of Ischemic Heart Disease

**DOI:** 10.3390/jcm10194530

**Published:** 2021-09-30

**Authors:** Takashi Hiraga, Yuichi Saito, Naoto Mori, Kazuya Tateishi, Hideki Kitahara, Yoshio Kobayashi

**Affiliations:** 1 Department of Cardiovascular Medicine, Chiba University Graduate School of Medicine, Chiba 260-0856, Japan; t_hiraga1990@yahoo.co.jp (T.H.); kazuyatateishi0926@gmail.com (K.T.); hidekita.0306@gmail.com (H.K.); yuiryosuke@msn.com (Y.K.); 2 Department of Internal Medicine, Chiba Aoba Municipal Hospital, Chiba 260-0852, Japan; polymol.3321@gmail.com

**Keywords:** uric acid, endothelial function, ischemic heart disease, ischemia with no obstructive coronary artery disease

## Abstract

Previous studies indicated that serum uric acid (SUA) level is a marker of endothelial function in subsets of ischemic heart disease (IHD). In the present study, we aimed to evaluate the relation between the SUA level and endothelial function in patients with a broad spectrum of IHD, including obstructive coronary artery disease (CAD) and ischemia with no obstructive CAD (INOCA). Three prospective studies and one retrospective study were pooled, in which the SUA level was measured, and systemic endothelial function was assessed using the reactive hyperemia index (RHI). The primary endpoint of the present study was a correlation of the SUA level with RHI. A total of 181 patients with a broad spectrum of IHD were included, among whom, 46 (25%) had acute coronary syndrome presentation and 15 (8%) had INOCA. Overall, the SUA level was negatively correlated with the RHI (r = −0.22, *p* = 0.003). Multivariable analysis identified the SUA level and INOCA as significant factors associated with RHI values. In conclusion, in patients with a broad spectrum of IHD, including obstructive epicardial CAD (chronic and acute coronary syndromes) and INOCA, the SUA level was significantly and negatively correlated with systemic endothelial function assessed with the RHI. INOCA, rather than obstructive CAD, was more associated with endothelial dysfunction.

## 1. Introduction

Angina is a common clinical presentation of ischemic heart disease (IHD), which affects more than 100 million people worldwide [1]. The traditional understanding of IHD includes chronic coronary syndrome (CCS) and acute coronary syndrome (ACS) due to epicardial coronary artery narrowings and occlusions [2]. ACS is a part of the natural history of CCS, but from a clinical perspective, the two entities are different [3]. A large US registry showed that approximately one third of patients undergoing elective coronary angiography for the investigation of angina do not have obstructive epicardial coronary artery disease (CAD) [4], suggesting ischemia with no obstructive CAD (INOCA) accounts for a sizable proportion in IHD.

Uric acid, the end-product of purine metabolism in humans, is associated with inflammation, oxidative stress, and endothelial dysfunction, contributing to the development of atherosclerotic diseases including IHD [5]. Previous studies have indicated that urate lowering therapy had an effect on blood pressure and endothelial function [5], and we and others have reported that the serum uric acid (SUA) level was a marker or predictor of systemic endothelial dysfunction in patients with ACS and INOCA [6,7,8]. However, whether the SUA level is associated with impaired endothelial function in patients with a broad spectrum of IHD is unclear. Additionally, the impact of a subset of IHD on endothelial dysfunction remains unknown. The aim of the present study was to evaluate the relation between the SUA level and systemic endothelial function in patients with obstructive CAD (CCS and ACS) undergoing percutaneous coronary intervention (PCI) and coronary artery bypass grafting (CABG), and INOCA.

## 2. Methods

### 2.1. Study Population and Definitions

**Figure 1:**
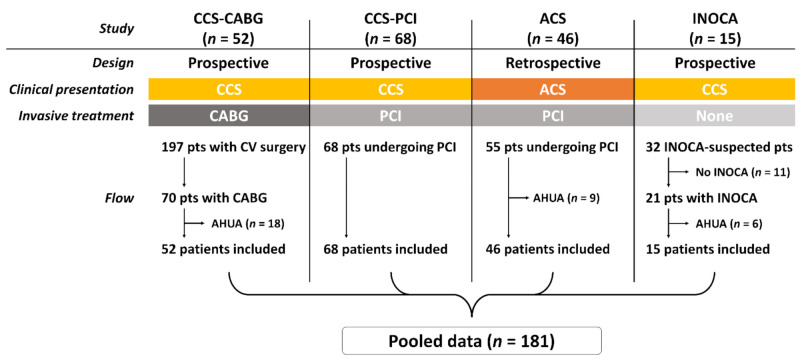
Study flow. ACS: acute coronary syndrome, AHUA: antihyperuricemic agent, CABG: coronary artery bypass grafting, CCS: chronic coronary syndrome, INOCA: ischemia with no obstructive coronary artery disease, PCI: percutaneous coronary intervention.

We have conducted three prospective studies and one retrospective study to evaluate systemic endothelial function at Chiba University Hospital, in which patients with various types of IHD were included. The present study was a post hoc analysis using pooled data of the four studies (Figure 1). All studies were approved by the institutional ethics committee and conducted in accordance with the Declaration of Helsinki. Each study included patients (1) undergoing elective CABG procedures, (2) undergoing elective PCI, (3) with ACS who underwent PCI, and (4) with INOCA including vasospastic angina (VSA) and/or microvascular dysfunction (MVD). In all studies, SUA levels were measured at baseline and systemic endothelial function was non-invasively assessed. Individual patient data were pooled to create the dataset and to evaluate the impact of SUA level on endothelial dysfunction in patients with a broad spectrum of IHD. Patients with antihyperuricemic agents were excluded.

A study (*n* = 197) enrolled patients planned for elective cardiovascular surgery, among whom, 70 underwent isolated or concomitant CABG procedures (registered at the University Hospital Medical Information Network Clinical Trials Registry: UMIN000015135) [9,10]. After excluding 18 patients who received antihyperuricemic agents, 52 were included in the present analysis (CCS-CABG group). Another CCS cohort included 68 patients undergoing elective PCI procedures under intravascular ultrasound guidance (UMIN000027855) (CCS-PCI group) [11,12]. Patients with ACS (*n* = 46) were also included in the present pooled data from a retrospective study (ACS group) [6]. All patients in the CCS-PCI and ACS groups underwent PCI procedures per local standard practice. Patients received dual antiplatelet therapy before or at the time of PCI, and radial artery approach, intracoronary imaging, and contemporary drug-eluting stents were predominantly used [13,14,15,16,17,18]. The fourth study was a prospective investigation in which intracoronary acetylcholine (ACh) provocation tests and invasive wire-based physiological assessment were employed to diagnose vasospastic angina and/or coronary microvascular dysfunction in patients with suspected INOCA (UMIN000019863) [8,19]. INOCA was defined as having a positive ACh provocation test (angiographic coronary artery vasospasm accompanied by chest pain or ischemic electrocardiographic changes) and/or microvascular dysfunction (coronary flow reserve ≤ 2.5 or index of microcirculatory resistance ≥ 25) [8,19]. A total of 15 patients with INOCA (vasospastic angina and/or microvascular dysfunction) were included in the present pooled data (INOCA group).

Hypertension was defined as having a previous diagnosis of hypertension or previous antihypertensive medications. Diabetes mellitus was defined as a previous diagnosis of diabetes or previous glucose lowering medications, or hemoglobin A1c ≥ 6.5%. Dyslipidemia was defined as low-density lipoprotein cholesterol ≥ 140 mg/dL, high-density lipoprotein cholesterol < 40 mg/dL, or fasting triglycerides > 150 mg/dL, or a previous diagnosis of dyslipidemia. Current smoking was defined as a history of smoking within the past year [20]. In addition, hyperuricemia was defined as >7 mg/dL for men and >6 mg/dL for women. Estimated glomerular filtration rate was calculated with the modification of diet in renal disease equation using the Japanese coefficient according to the Kidney Disease Outcomes Quality Initiative clinical guidelines [21].

### 2.2. Endothelial Function Assessment

Systemic endothelial function was assessed with reactive hyperemic index (RHI) using the EndoPAT 2000 device (Itamar Medical Inc., Caesarea, Israel), which is validated to evaluate endothelial function non-invasively, operator-independently, and reproducibly [22]. RHI was measured as previously described [6,8,9,10,11,12,19]. Briefly, patients fasted and refrained from taking caffeine, tobacco, and all medications for at least eight hours. RHI was measured in a quiet and temperature-controlled room in the early morning. The dedicated probes to measure arterial pulse wave were placed on the index fingers and a blood pressure cuff was placed on either upper arm. The baseline pulse amplitude was evaluated for the first 5-min period. The cuff was subsequently inflated for five minutes, and then deflated to induce reactive hyperemia for the next five minutes. The EndoPAT 2000 device automatically calculated RHI, which is the ratio of amplitude of arterial pulse wave after deflation period divided by those before inflation period, indexed to the contralateral arm. RHI was measured before the invasive procedures (i.e., CABG, PCI, and intracoronary diagnostic investigations) in the CCS-CAGB, CCS-PCI, and INOCA groups, while in the ACS group, endothelial function was evaluated on the day of discharge or 1 day earlier [6]. Patients were divided into two groups according to a cut-off value of RHI of 1.67 [23].

### 2.3. Endpoint and Statistical Analysis

The primary endpoint of the present pooled study was a correlation of SUA level with endothelial function assessed with RHI. The impact of a subset in a broad spectrum of IHD on RHI was also evaluated. Exploratory analysis on clinical outcomes was performed to identify major adverse cardiovascular events (MACE), a composite of all-cause death, myocardial infarction, and stroke. Statistical analysis was performed with SAS software version 9.3 (SAS Institute, Cary, NC, USA). All data are expressed as mean ± standard deviation, median (interquartile range), or frequency (%), as appropriate. Continuous variables were compared with Student’s *t*-test and analysis of variance, and categorical variables were assessed with Fisher’s exact test. A normal distribution was visually assessed with P-P plots and was tested using Kolmogorov–Smirnov test. The correlation between variables were analyzed using Pearson’s correlation coefficient. Kaplan–Meier analysis with the log-rank test was employed to assess MACE-free survival rates. Age, sex, and factors associated with variables on univariable analyses (*p* < 0.10) were included into multivariable analysis. Multiple linear regression analysis was performed to identify factors associated with RHI, and multivariable logistic regression analysis for RHI < 1.67 was also conducted as a sensitivity analysis, presented as odds ratio with 95% confidence intervals. A value of *p* < 0.05 was considered statistically significant.

## 3. Results

A total of 181 patients with a broad spectrum of IHD were included, of whom, 46 (25%) had ACS presentation and 15 (8%) had no obstructive epicardial CAD (i.e., INOCA) (Figure 1). Hyperuricemia was observed in 26 (19%) and 10 (22%) men and women (*p* = 0.83). 

Table 1 and Table S1 list the overall baseline characteristics and those among the four groups. The mean SUA level was 5.7 ± 1.5 mg/dL, and RHI < 1.67 was observed in 75 (41%) patients.

**Figure 2:**
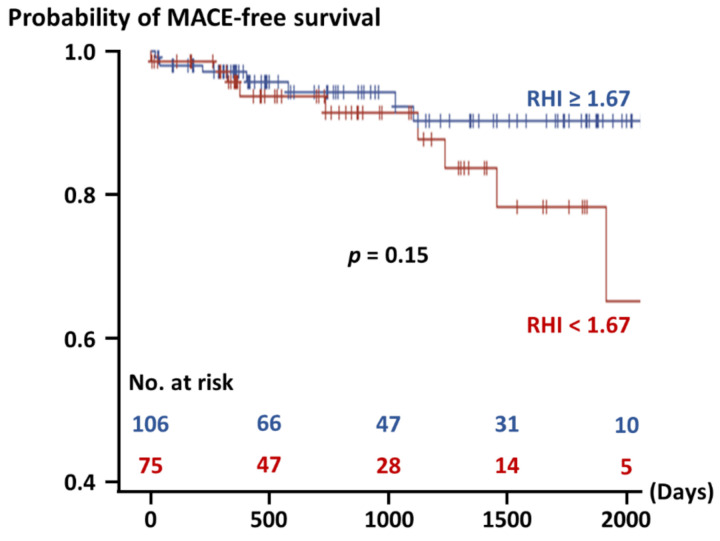
Probability free from major adverse cardiovascular events. MACE: major adverse cardiovascular events, RHI: reactive hyperemia index.

Overall, the SUA level was negatively correlated with the RHI (r = −0.22, *p* = 0.003). Multivariable analysis identified the SUA level and INOCA as significant factors associated with RHI values (Table 2). As a sensitivity analysis, logistic regression analysis confirmed the SUA level and INOCA as predictors of an RHI < 1.67 (Table S2). During the median follow-up period of 792 (362, 1540) days, 16 (8.8%) patients experienced MACE (Table S3). The patients with an RHI < 1.67 were non-significantly associated with an increased risk of MACE than those with an RHI ≥ 1.67 (Figure 2).

## 4. Discussion

The present study demonstrated that in patients with various types of IHD, including obstructive CAD (CCS and ACS) and INOCA, systemic endothelial dysfunction was found in 41%. The SUA level was significantly and negatively associated with the RHI. In addition to the SUA level, INOCA rather than other obstructive CAD was identified as a factor related to endothelial dysfunction in the present study population. To our knowledge, this is the first study investigating systemic endothelial function in a broad spectrum of IHD.

The current guidelines for the diagnosis and management of IHD are predominantly shaped by the burden of epicardial obstructive CAD, including CCS and ACS [3,24], while recent investigations have shown that a sizable proportion of patients with angina have no obstructive epicardial CAD but myocardial ischemia, namely INOCA [25]. Consequently, consensus documents have been published to provide definitions and guidance on the diagnostic approach and management of INOCA [26,27]. The European consensus document indicated that vasospastic angina and coronary microvascular dysfunction are the major endotypes of INOCA, and the present study determined both as the definition of the INOCA group. It is well known that endothelial dysfunction plays important roles in the development of obstructive CAD and INOCA [27,28]. However, few studies have investigated the relation of endothelial function to the entire spectrum of IHD. In this context, the present study confirmed that endothelial function is a key underlying mechanism in IHD. Interestingly, INOCA rather than obstructive CAD was identified as a factor strongly associated with systemic endothelial dysfunction in this study. Given that coronary vasospasm is not necessarily provoked in patients with established obstructive CAD when an ACh provocation test is performed, this finding may be reasonable and be translated into diagnostic and therapeutic approaches for INOCA. As indicated in the recent European guidelines (Class IIb) [3], an endothelium-dependent diagnostic procedure (i.e., an ACh provocation test) may be considered in suspected INOCA. Whether endothelium targeting therapy improves clinical outcomes and quality of life in INOCA remains largely unknown but deserves further investigation.

We and other groups have previously shown the SUA level as a marker or predictor of systemic endothelial dysfunction in patients with ACS and INOCA [6,7,8], and the present study supported the concept in the entire spectrum of IHD. While numerous epidemiological studies have reported the association of the SUA level with cardiovascular disease, including IHD [5], a therapeutic intervention targeting SUA in IHD has not been fully investigated. Noman et al. previously demonstrated that in CCS patients with angiographically confirmed obstructive CAD, allopurinol (600 mg/day) significantly improved exercise capacity in a randomized, placebo-controlled, cross-over setting [29]. Although it is unclear whether xanthin oxidase inhibition itself or the reduced SUA levels by allopurinol (or both) prolonged the exercise time, the improvement in peripheral and coronary endothelial function were suggested as the mechanism in their paper [29]. Despite the modest correlation (r = −0.22, *p* = 0.003), SUA lowering therapy might be beneficial in patients with IHD.

The present study has several limitations. The present pooled data consist of three prospective studies and one retrospective study and were assessed as a post hoc analysis. ACS presentation accounted for 28% among the patients with obstructive CAD, which is in line with current clinical practice in Japan, while only 8% of the patients had INOCA in the present study. Given that 30–50% of patients may reportedly have INOCA among those undergoing invasive coronary angiography [4,25], the impact of INOCA may have been underrepresented in the present pooled data. Nevertheless, multivariable analyses identified INOCA as a significant factor associated with a lower RHI, reinforcing a crucial role of systemic endothelial dysfunction in INOCA. Despite the multivariable analyses, there were several confounding factors and unmeasured variables including medications and detailed data on blood pressure and a smoking habit (e.g., 24-h ambulatory blood pressure monitoring, and number of cigarettes smoked per day). Although a meta-analysis reported the prognostic impact of the RHI [30], clinical outcomes were not significantly different between the patients with endothelial dysfunction (RHI < 1.67) and their counterpart in the present study, probably because of the small sample size.

## 5. Conclusions

In patients with a broad spectrum of IHD, including obstructive CAD (CCS and ACS) and INOCA, the SUA level was significantly and negatively correlated with systemic endothelial function assessed with the RHI. INOCA, rather than obstructive CAD, was more associated with endothelial dysfunction.

## Figures and Tables

**Table 1:** Baseline characteristics.

Variable	All
	(*n* = 181)
Age (years)	68.9 ± 10.9
Men	135 (75%)
Body mass index (kg/m^2^)	24.0 ± 3.9
Hypertension	133 (73%)
Diabetes mellitus	73 (40%)
Dyslipidemia	129 (71%)
Current smoker	44 (24%)
Prior myocardial infarction	40 (22%)
eGFR (ml/min/1.73 m^2^)	67.2 ± 19.9
Serum uric acid (mg/dL)	5.7 ± 1.5
LDL cholesterol (mg/dL)	107.5 ± 34.6
HDL cholesterol (mg/dL)	52.2 ± 16.0
Non-fasting triglyceride (mg/dL)	136.7 ± 80.7
Hemoglobin A1c (%)	6.4 ± 1.3
Clinical presentation	
Acute coronary syndrome	46 (25%)
Chronic coronary syndrome	135 (75%)
Medical treatment	
Aspirin	111 (61%)
P2Y12 inhibitor	56 (31%)
Oral hypoglycemic agent	48 (27%)
Metformin	20 (11%)
SGLT2 inhibitor	7 (4%)
ACE-I or ARB	90 (50%)
β-blocker	56 (31%)
Calcium channel blocker	86 (48%)
Diuretic	32 (18%)
Statin	108 (60%)
Fibrate	2 (1%)
Reactive hyperemia index	1.84 ± 0.53

ACE-I: angiotensin converting enzyme inhibitor, ARB: angiotensin II receptor blocker, eGFR: estimate glomerular filtration rate, HDL: high density lipoprotein, LDL: low density lipoprotein, SGLT: sodium-glucose cotransporter.

**Table 2:** Predictors of reactive hyperemia index.

Variable	Univariable		Multivariable	
	r	*p* Value	β	*p* Value
Age (years)	0.09	0.20	−0.01	0.94
Men	−0.03	0.65	0.004	0.95
Body mass index (kg/m^2^)	−0.13	0.09	−0.07	0.37
Hypertension	−0.02	0.83		
Diabetes mellitus	−0.06	0.45		
Dyslipidemia	0.01	0.91		
Current smoker	−0.14	0.07	−0.09	0.25
Prior myocardial infarction	−0.02	0.82		
eGFR (ml/min/1.73 m^2^)	0.12	0.11		
Serum uric acid (mg/dL)	−0.22	0.003	−0.22	0.004
LDL cholesterol (mg/dL)	0.003	0.97		
HDL cholesterol (mg/dL)	0.08	0.28		
Non-fasting triglyceride (mg/dL)	−0.07	0.33		
Hemoglobin A1c (%)	−0.04	0.63		
INOCA	−0.15	0.04	−0.16	0.03

eGFR: estimated glomerular filtration rate, HDL: high density lipoprotein, INOCA: ischemia with no obstructive coronary artery disease, LDL: low density lipoprotein.

## Data Availability

Data sharing not applicable.

## Supplementary Materials

The following are available online at https://www.mdpi.com/article/10.3390/jcm10194530/s1, Table S1: Baseline characteristics; Table S2: Predictors of reactive hyperemia index < 1.67; Table S3: Clinical outcomes.
