# Supplementary material for: Impact of Serum Uric Acid Level on Systemic Endothelial Dysfunction in Patients with a Broad Spectrum of Ischemic Heart Disease
Source: J Clin Med. 2021 Sep 30;10(19):4530. doi: 10.3390/jcm10194530 (PMC8509425; doi:10.3390/jcm10194530)
Supplement: Supplementary file 1 [file jcm-10-04530-s001.zip › jcm-1388613-supplementary/Table S1-3/Table S1.pdf]

Table S1. Baseline characteristics

Variable	CABG (n=52)	PCI (n=68)	ACS (n=46)	INOCA (n=15)	P value
Age (years)	70.5±8.9	70.3±10.8	66.0±12.0	65.7±13.0	0.07
Men	38 (73%)	57 (84%)	33 (72%)	7 (47%)	0.03
Body mass index (kg/m^2)	23.4±3.6	24.3±3.8	24.0±4.1	25.1±4.8	0.44
Hypertension	42 (81%)	46 (68%)	35 (76%)	10 (67%)	0.37
Diabetes mellitus	27 (52%)	31 (46%)	13 (28%)	2 (13%)	0.01
Dyslipidemia	36 (69%)	48 (71%)	36 (78%)	9 (60%)	0.53
Current smoker	11 (21%)	12 (18%)	16 (35%)	5 (33%)	0.14
Prior myocardial infarction	16 (31%)	19 (28%)	5 (11%)	0 (0%)	0.006
eGFR (ml/min/1.73 m^2)	56.7±20.6	71.5±17.8	72.3±19.4	69.0±15.5	<0.001
Serum uric acid (mg/dl)	6.0±1.6	5.5±1.5	5.7±1.5	5.3±0.9	0.24
LDL cholesterol (mg/dl)	111.6±34.4	92.4±26.3	122.4±38.9	119.5±29.0	<0.001
HDL cholesterol (mg/dl)	53.2±14.7	51.9±16.4	49.0±14.0	59.9±22.2	0.14
Non-fasting triglyceride (mg/dl)	123.4±73.1	139.6±65.5	134.6±60.8	175.8±171.6	0.17
Reactive hyperemia index	1.84±0.49	1.86±0.52	1.91±0.52	1.57±0.39	0.20
Hemoglobin A1c (%)	6.4±1.0	6.3±1.1	6.5±2.0	6.0±0.5	0.12

eGFR: estimate glomerular filtration rate, HDL: high density lipoprotein, LDL: low density lipoprotein.
