# Supplementary material for: Impact of Serum Uric Acid Level on Systemic Endothelial Dysfunction in Patients with a Broad Spectrum of Ischemic Heart Disease
Source: J Clin Med. 2021 Sep 30;10(19):4530. doi: 10.3390/jcm10194530 (PMC8509425; doi:10.3390/jcm10194530)
Supplement: Supplementary file 1 [file jcm-10-04530-s001.zip › jcm-1388613-supplementary/Table S1-3/Table S2.pdf]

Table S2. Predictors of reactive hyperemia index <1.67

Variable	Univariable		Multivariable	
	OR (95% CI)	P value	OR (95% CI)	P value
Age (years)	0.99 (0.97-1.02)	0.52	1.00 (0.97-1.03)	0.86
Men	1.01 (0.51-1.99)	0.98	1.00 (0.47-2.10)	0.99
Body mass index (kg/m^2)	1.07 (0.99-1.16)	0.08	1.04 (0.95-1.14)	0.39
Hypertension	0.99 (0.51-1.93)	0.97		
Diabetes mellitus	0.89 (0.49-1.63)	0.70		
Dyslipidemia	1.19 (0.62-2.30)	0.61		
Current smoker	1.10 (0.55-2.19)	0.79		
Prior myocardial infarction	1.37 (0.68-2.78)	0.38		
eGFR (ml/min/1.73 m^2)	0.99 (0.98-1.01)	0.21		
Serum uric acid (mg/dl)	1.25 (1.02-1.54)	0.03	1.28 (1.03-1.60)	0.03
LDL cholesterol (mg/dl)	1.00 (0.99-1.01)	0.98		
HDL cholesterol (mg/dl)	0.99 (0.98-1.01)	0.55		
Non-fasting triglyceride (mg/dl)	1.00 (1.00-1.01)	0.06	1.00 (1.00-1.01)	0.14
Hemoglobin A1c (%)	1.06 (0.85-1.33)	0.60		
INOCA	3.11 (1.02-9.50)	0.047	3.24 (1.00-10.48)	0.049

CI: confidence interval, eGFR: estimated glomerular filtration rate, HDL: high density lipoprotein, INOCA: ischemia with no obstructive coronary artery disease, LDL: low density lipoprotein, OR: odds ratio.
