# Supplementary material for: Impact of Serum Uric Acid Level on Systemic Endothelial Dysfunction in Patients with a Broad Spectrum of Ischemic Heart Disease
Source: J Clin Med. 2021 Sep 30;10(19):4530. doi: 10.3390/jcm10194530 (PMC8509425; doi:10.3390/jcm10194530)
Supplement: Supplementary file 1 [file jcm-10-04530-s001.zip › jcm-1388613-supplementary/Table S1-3/Table S3.pdf]

**Table S3. Clinical outcomes**

| Variable | All (n=181) | RHI ≤1.67 (n=75) | RHI >1.67 (n=106) | P value |
|---|---|---|---|---|
| MACE | 16 (8.8%) | 9 (12.0%) | 7 (6.6%) | 0.29 |
| Death | 11 (6.1%) | 6 (8.0%) | 5 (4.7%) | 0.53 |
| Myocardial infarction | 4 (2.2%) | 2 (2.7%) | 2 (1.9%) | 1.00 |
| Stroke | 4 (2.2%) | 3 (4.0%) | 1 (0.9%) | 0.31 |

MACE: major adverse cardiovascular events, RHI: reactive hyperemia index.
